# Cytochrome P450 1B1 inhibition suppresses tumorigenicity of prostate cancer via caspase-1 activation

**DOI:** 10.18632/oncotarget.16598

**Published:** 2017-03-27

**Authors:** Inik Chang, Yozo Mitsui, Seul Ki Kim, Ji Su Sun, Hye Sook Jeon, Jung Yun Kang, Nam Ju Kang, Shinichiro Fukuhara, Ankurpreet Gill, Varahram Shahryari, Z. Laura Tabatabai, Kirsten L. Greene, Rajvir Dahiya, Dong Min Shin, Yuichiro Tanaka

**Affiliations:** ^1^ Department of Oral Biology, Yonsei University College of Dentistry, Seoul, South Korea; ^2^ Department of Surgery and Division of Urology, Veterans Affairs Medical Center, San Francisco, California, United States of America; ^3^ Department of Urology, University of California, San Francisco, California, United States of America; ^4^ BK21 PLUS Project, Yonsei University College of Dentistry, Seoul, South Korea; ^5^ Department of Pathology, Veterans Affairs Medical Center and University of California, San Francisco, California, United States of America

**Keywords:** CYP1B1, prostate cancer, tumorigenicity, shRNA, caspase-1

## Abstract

Cytochrome P450 1B1 (CYP1B1) is recognized as a universal tumor biomarker and a feasible therapeutic target due to its specific overexpression in cancer tissues. Despite its up-regulation in prostate cancer (PCa), biological significance and clinicopathological features of CYP1B1 are still elusive. Here, we show that overexpression or hyperactivation of CYP1B1 stimulated proliferative, migratory and invasive potential of non-tumorigenic PCa cells. Attenuation of CYP1B1 with its specific small hairpin (sh) RNAs greatly reduced proliferation through apoptotic cell death and impaired migration and invasion in PCa cells. Intratumoral injection of CYP1B1 shRNA attenuated growth of pre-existing tumors. The antitumor effect of CYP1B1 shRNA was also observed in prostate tumor xenograft mouse models. Among the genes altered by CYP1B1 knockdown, reduction of caspase-1 (CASP1) activity attenuated the antitumor effect of CYP1B1 inhibition. Indeed, CYP1B1 regulates CASP1 expression or activity. Finally, CYP1B1 expression was increased in higher grades of PCa and overall survival was significantly reduced in patients with high levels of CYP1B1 protein. CYP1B1 expression was reversely associated with CASP1 expression in clinical tissue samples. Together, our results demonstrate that CYP1B1 regulates PCa tumorigenesis by inhibiting CASP1 activation. Thus, the CYP1B1-CASP1 axis may be useful as a potential biomarker and a therapeutic target for PCa.

## INTRODUCTION

The cytochrome P450 (CYP) superfamily is involved in the metabolism of a diverse range of xenobiotics and endogenous compounds. Among CYP1 family members, CYP1B1 is the only member of the CYP1B subfamily and mainly implicated in the hydroxylation of estrogen and activation of environmental carcinogens [1]. CYP1B1 protein is found in the cancer cells of various cancerous tissues but is undetectable or minimally expressed in the adjacent normal cells of cancer tissues and normal tissues [2]. Therefore, CYP1B1 has been recognized as a potential tumor biomarker and a promising target for anticancer therapy [1].

Tumor-specific overexpression of CYP1B1 indicates its role as a regulator of tumor progression. Saini *et al*. showed that CYP1B1 knockdown inhibits endometrial carcinogenesis by affecting cellular proliferation, cell cycle and invasive potential through the regulation of cyclin E1, Skp2, and TRAIL [3]. In squamous cell carcinoma of the head and neck, CYP1B1 knockdown reduced the migration and proliferation of premalignant cells and CYP1B1-mediated estrogen metabolism is essential for cancer development [4]. We reported that CYP1B1 expression is regulated by miR-200c and high CYP1B1 levels contribute to resistance of renal cell carcinoma (RCC) to docetaxel [5]. In addition, CYP1B1 reduction altered expression of CDC20 and DAPK1 and resulted in the disturbance of cell cycle and apoptosis in RCC [6]. Recently, Kwon *et al*. suggested that CYP1B1-mediated Sp1 induction was critical for epithelial-mesenchymal transition and Wnt/β-catenin signaling in breast cancer [7].

Caspase-1 (CASP1) was originally characterized as cleaving inactive pro-interleukin (IL)-1β to produce active IL-1β and is considered an initiator caspase [8]. In addition to pro-inflammatory role, CASP1 can execute programmed cell death [9]. Activation of CASP1 is involved in caspase-3 activation and apoptosis induced by brain ischemia [10, 11]. It is required for apoptosis in Rat-1 fibroblasts as well as other mammalian and insect cells [8, 12]. In line with this evidence, CASP1 acts as a tumor suppressor regulating proliferation and apoptosis of epithelial cells. CASP1-deficiency has been found to enhance tumor formation in mouse colorectal cancer models [13, 14]. It is also frequently downregulated in prostate cancer (PCa) indicating its loss is a potential step in malignant progression [15, 16]. Additionally, CASP1 activation is required for human PCa cells to undergo apoptosis in response to transforming growth factor-β [17].

PCa, the most frequently diagnosed malignancy among males in the United States, accounted for an estimated 180,890 new cases and 26,120 deaths due to PCa in 2016 [18]. The majority of localized and androgen-dependent prostate cancers are generally curative by radical prostatectomy, radiotherapy, hormonal therapy, and/or neoadjuvant chemotherapy. However, no effective therapies are available for androgen-independent disease and metastatic PCa [19, 20].

CYP1B1 expression has been found in both normal prostate tissues and prostate tumors including normal-adjacent tissues, with markedly higher levels in PCa compared with benign tissues [21, 22]. CpG methylation of the promoter/enhancer has been suggested as a mechanism controlling cancer-specific expression of CYP1B1 [22]. CYP1B1 metabolites such as 4-hydroxyestradiol and 2-amino-1-methyl-6-phenylimidazo[4,5-b]pyridine were reported to induce PCa in experimental animal models [23, 24]. Exposure of rat prostate epithelial cells to the metabolites also results in DNA damage and neoplastic changes implicating its carcinogenic effects in the prostate [25]. Polymorphic variants of CYP1B1 altering the catabolism of estrogen may modify prostate cancer risk and may also predict response to chemotherapy [26]. These evidences strongly suggest that CYP1B1 plays a potentially crucial role in prostate tumor development and progression. However, the functional impact and clinicopathological significance of CYP1B1 up-regulation in PCa are still largely unknown.

In this study, we explored the functional role of CYP1B1 in the transformation and tumorigenesis of PCa. We also assessed the feasibility of CYP1B1 targeting for the treatment of PCa in an experimental animal model. The molecular target of CYP1B1 action and clinical relevance of its overexpression were also elucidated.

## RESULTS

### CYP1B1 overexpression stimulates tumorigenesis in prostate epithelial cells

**Figure 1:**
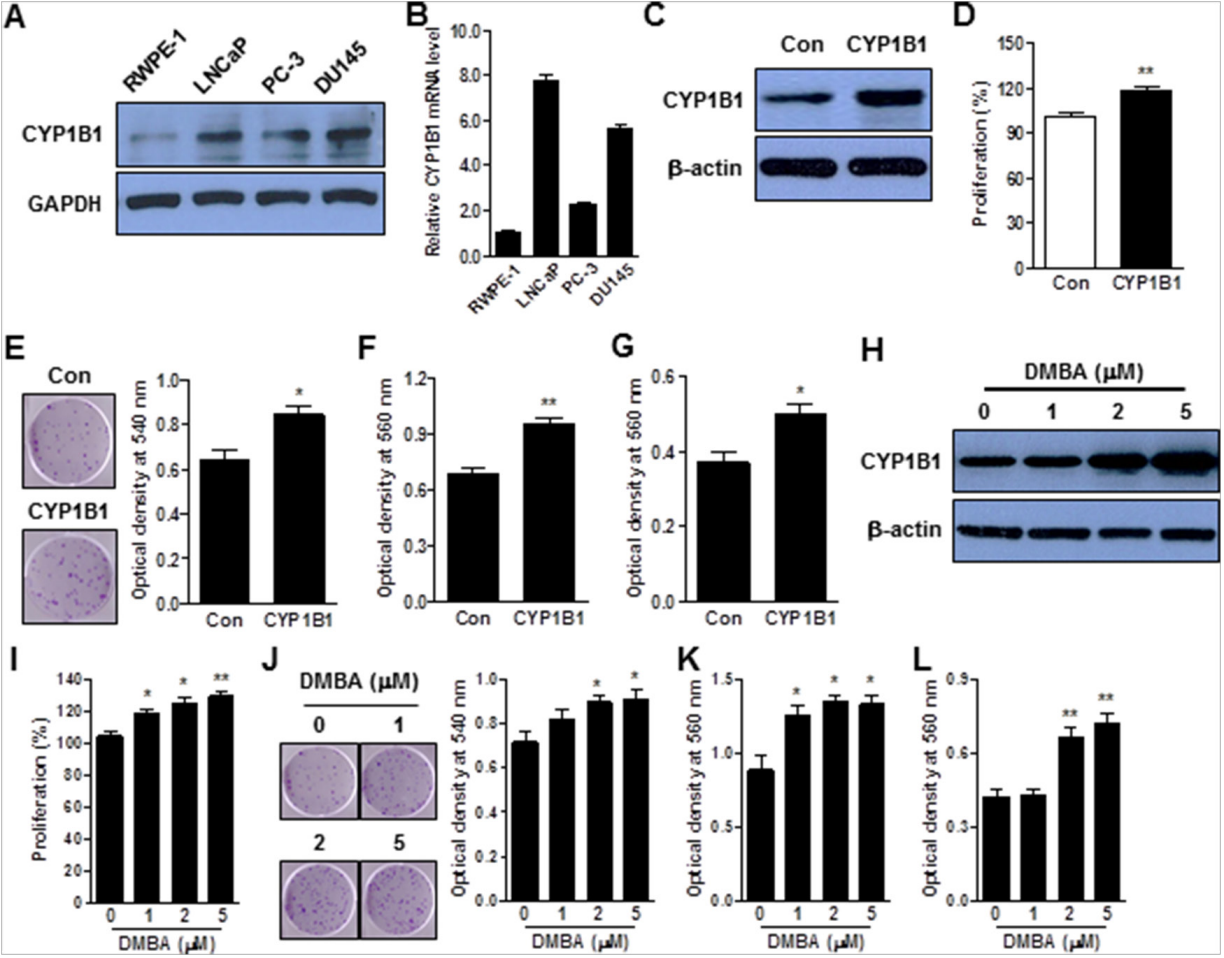
CYP1B1 promotes cellular transformation of RWPE-1 cells **(A and B)** Endogenous expression of CYP1B1 protein (A) and mRNA (B) in PCa cells. Levels were determined by Western blot and qRT-PCR, respectively. **(C–G)** Effect of CYP1B1 overexpression on *in vitro* tumorigenicity. Ectopic expression of CYP1B1 in transfected RWPE-1 cells as examined by Western blot (C). Cell proliferation as determined by MTS assay (D). Colony formation as determined by crystal violet staining. Representative image of colonies (left panel) and quantification of stained colonies (right panel) are shown (E). Cell migration (F) and invasion (G) capability as determined by transwell migration and invasion assay, respectively. **(H–L)** Effect of DMBA treatment on *in vitro* tumorigenicity. Induction of CYP1B1 expression in RWPE-1 cells as determined by Western blot (H). Cell proliferation as determined by MTS assay (I). Colony formation as determined by crystal violet staining. Representative image of colonies (left panel) and quantification of stained colonies (right panel) are shown (J). Cell migration (K) and invasion (L) capability as determined by transwell migration and invasion assay, respectively. *p<0.05; **p<0.01.

CYP1B1 expression was examined in human prostate cell lines, which included a non-tumorigenic prostate epithelial cell line (RWPE-1) and prostate carcinoma cell lines (LNCaP, PC-3 and DU145). Relative expression of CYP1B1 protein (Figure 1A) and mRNA (Figure 1B) was amplified in all the examined cancer cell lines compared with the RWPE-1 cells. To explore the functional role of CYP1B1 in PCa, we first investigated the effect of its up-regulation in prostate epithelial cells using RWPE-1 cell lines. Exogenous expression of CYP1B1 (Figure 1C) significantly enhanced proliferation and colony formation (Figure 1D and 1E) and also stimulated cell motility and invasiveness of RWPE-1 cells (Figure 1F and 1G). In addition, DMBA, a chemical activator of CYP1B1 induced CYP1B1 expression in a dose-dependent manner (Figure 1H) and promoted growth, motility and invasiveness of RWPE-1 cells (Figure 1I to 1L). These results indicate that overexpression and activation of CYP1B1 regulates oncogenic activity in prostate cells.

### CYP1B1 inhibition suppresses PCa tumorigenesis *in vitro*

**Figure 2:**
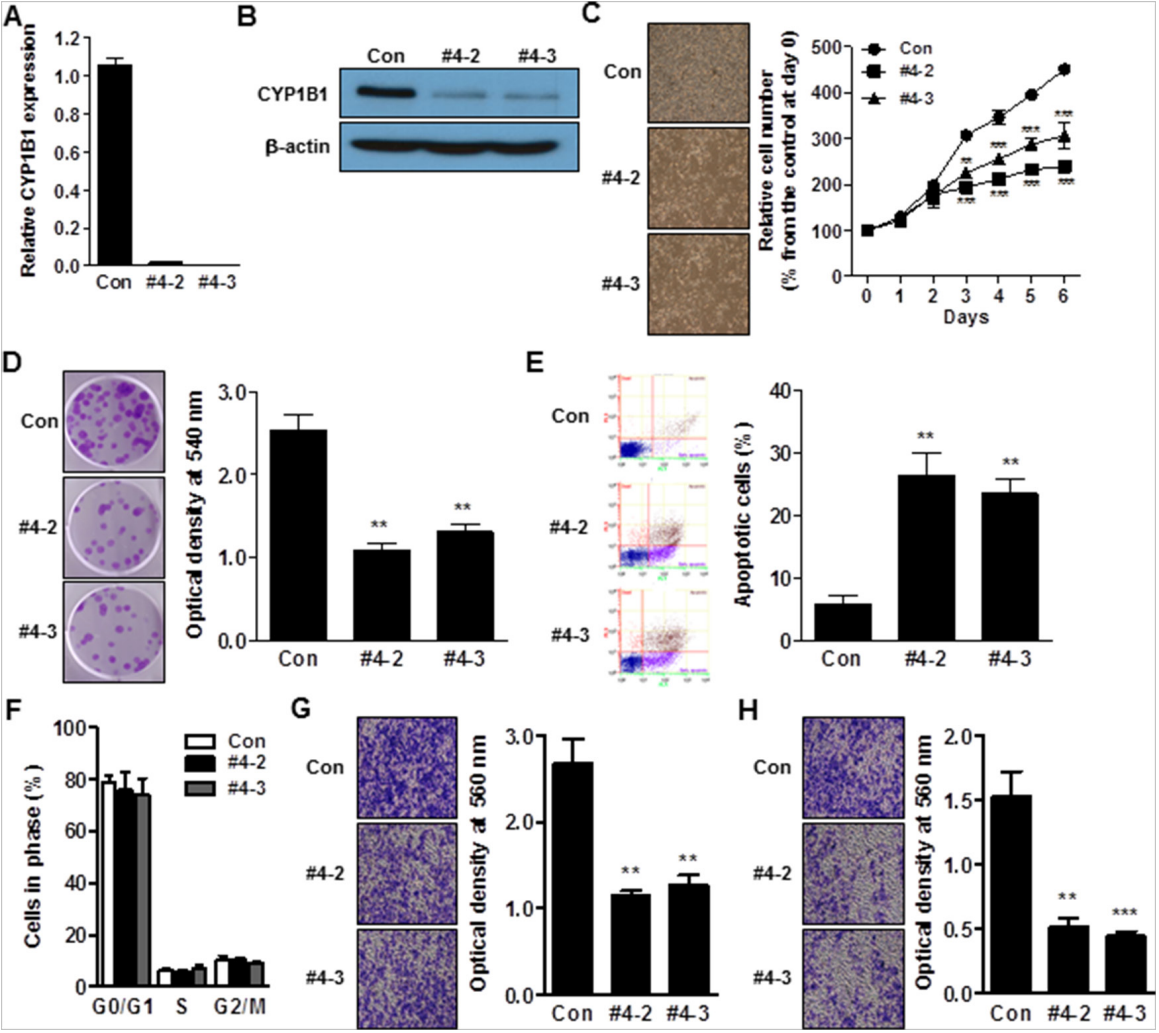
CYP1B1 inhibition suppresses *in vitro* tumorigenicity **(A and B)** Expression of CYP1B1 mRNA (A) and protein (B) in CYP1B1 shRNA or control shRNA expressing PC-3 cells. Levels were determined by qRT-PCR and Western blot, respectively. **(C–H)** Effect of CYP1B1 knockdown on *in vitro* tumorigenicity. Cell proliferation as determined by MTS assay at the indicated times. Representative images of cell morphology (left panel) and quantification of cell proliferation (right panel) are shown (C). Colony formation as determined by crystal violet staining. Representative image of colonies (left panel) and quantification of stained colonies (right panel) are shown (D). Apoptotic cell death as determined by flow cytometric analysis using double staining with Annexin V-FITC and 7-AAD. Representative biparametric histograms exhibiting cell (left panel) and quantification of apoptotic cells (right panel) are shown (E). Cell cycle progression as determined by DAPI staining (F). Cell migration (G) and invasion (H) capability as determined by transwell migration and invasion assay, respectively. Representative images (left panel) and quantification of assay (right panel) are shown. **p<0.01; ***p<0.001.

Since CYP1B1 is up-regulated in PCa cells, we used a CYP1B1 knockdown approach with lentivirus-mediated shRNA targeting CYP1B1 to explore its biological significance. To this end, we generated CYP1B1 deficient PC-3 and DU145 cells. Among the 4 shRNA candidates, the CYP1B1 shRNA #4 construct in PC-3 cells and #2 construct in DU145 cells exhibited the best efficiency by transient transfection and were used for establishing stable cell lines (Supplementary Figure 1). Subclones were produced from stably transfected CYP1B1 shRNAs and the resultant knockdown cell lines, PC-3/CYP1B1 shRNA #4-2 and #4-3, and DU145/CYP1B1 shRNA #2-12 and #2-23, were selected for the further functional studies (Figure 2A and 2B; Supplementary Figure 2A and 2B). No difference in cell growth and morphology was observed during first and the 2 days of cell growth but cell proliferation was significantly decreased starting 3 days after seeding of both CYP1B1 knockdown cell lines (Figure 2C and Supplementary Figure 2C). Clonogenic assays also confirmed the impact of CYP1B1 inhibition on delayed growth of cells (Figure 2D and Supplementary Figure 2D). To further understand the growth-inhibitory effect of CYP1B1 reduction, we assessed apoptosis and cell cycle distribution by flow cytometric analysis. The apoptotic cell fractions were significantly increased in CYP1B1 knockdown cells (Figure 2E and Supplementary Figure 2E). However, no significant change in cell cycle progression was observed (Figure 2F and Supplementary Figure 2F). We did observe that CYP1B1 inhibition results in lower motility and invasiveness of PCa cells (Figure 2G and 2H; Supplementary Figure 2G and 2H). These results suggest that CYP1B1 plays a role as an oncogene in PCa and its targeted inhibition could suppress tumorigenesis.

### CYP1B1 inhibition suppresses PCa tumorigenesis *in vivo*

**Figure 3:**
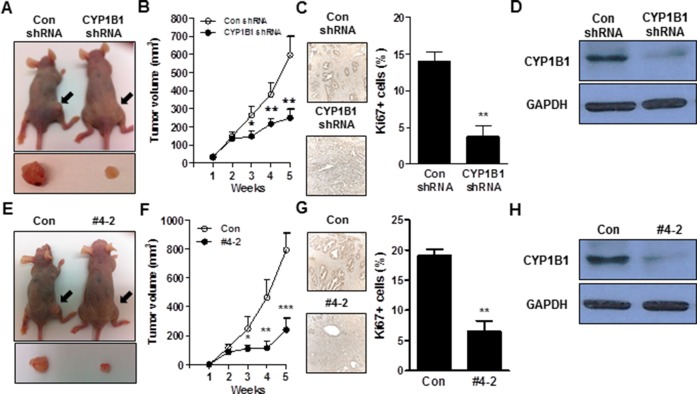
CYP1B1 inhibition suppresses *in vivo* tumor growth **(A to D)** Effect of intratumoral injection of CYP1B1 shRNA on *in vivo* tumor growth. Volume of tumor established by PC-3 cells after administration of shRNAs for CYP1B1 or control. Representative image of tumors grown in mice and tumors extracted from individual mice (A) and quantification of tumor volume (B). Quantification of Ki67-positive cells in the control or CYP1B1 shRNA injected tumors. Representative image of section (left panel) and quantification of stained cells (right panel) are shown. Total 20 fields/sample were counted (C). CYP1B1 protein expression in tumors injected with control or CYP1B1 shRNA (D). **(E–H)** Effect of CYP1B1 shRNA knockdown on *in vivo* tumor growth using xenograft mouse model. Volume of tumor established by PC-3 cells stably expressing CYP1B1 shRNA #4-2 in xenografts. Representative image of tumors grown in mice and tumors extracted from individual mice (E) and quantification of tumor volume (F). Quantification of Ki67-positive cells in the control or CYP1B1 shRNA #4-2 expressing xenograft tumors. Representative image of section (left panel) and quantification of stained cells (right panel) are shown. Total 20 fields/sample were counted (G). CYP1B1 protein expression of control or CYP1B1 shRNA #4-2 expressing xenograft tumors (H). *p<0.05; **p<0.01; ***p<0.001.

To validate the antitumor effect of CYP1B1 inhibition and further examine its use as therapeutic target for prostate cancer, *in vivo* models were utilized. In one model, CYP1B1 or control shRNA were administered intratumourally after allowing inoculated PC-3 cells to establish into tumors. As shown in Figure 3A and 3B, decreased growth was observed in tumors receiving CYP1B1 shRNA compared to those treated with control shRNA. The average tumor size after 5 weeks of control shRNA injection was 595.7±102.6 mm^3^ compared to 249.3±46.6 mm^3^ in tumors treated with CYP1B1 shRNA. Administration of CYP1B1 shRNA induced low cell proliferation in tumors compared to those treated with control shRNA (Figure 3C). Reduction of CYP1B1 protein level was confirmed in tumors injected with CYP1B1 shRNA (Figure 3D). In addition, PC-3 cells stably expressing CYP1B1 shRNA #4-2 or control shRNA were subcutaneously injected into the flank of nude mice. Decreased prostate tumor growth was observed in tumors expressing CYP1B1 shRNA #4-2 compared to those expressing control shRNA (Figure 3E). The average tumor size after 5 weeks was 792.5±116.2 mm^3^ in PC-3/control shRNA cells and 240.0±81.9 mm^3^ in tumors stably expressing CYP1B1 shRNA #4-2 (Figure 3F). Tumor cells containing CYP1B1 shRNA displayed a significant difference in the number of Ki67-positive cells compared to cells harboring control shRNA (Figure 3G). The level of CYP1B1 protein was reduced in tumors expressing CYP1B1 shRNA (Figure 3H). These results suggest that CYP1B1 inhibition effectively inhibits growth of tumor cells *in vivo* and could be a therapeutic target for PCa.

### Identification of a potential target of CYP1B1 inhibition-mediated anti-tumor activity

**Figure 4:**
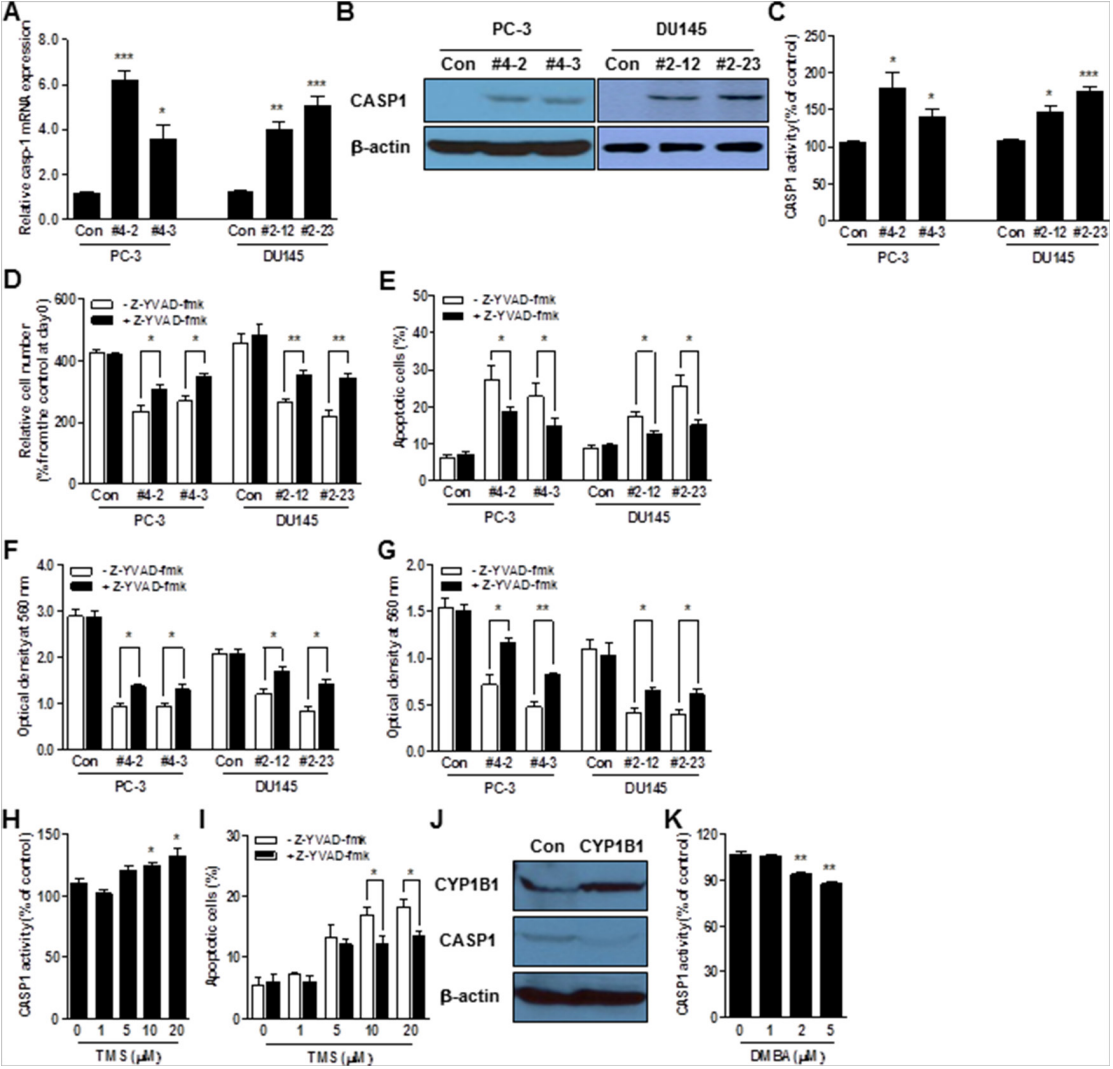
CASP1 is a functional target of CYP1B1 **(A–C)** CASP1 mRNA (A), protein (B), and enzyme activity (C) in CYP1B1 shRNA stably expressing PCa cells. Levels were determined by qRT-PCR, Western blot and colorimetric assay, respectively. **(D–G)** Effect of CASP1 inhibition on the tumorigenicity of CYP1B1 shRNA stably expressing PCa cells. Cells were treated with Z-YVAD-fmk (100 μM) and proliferation (D), apoptotic cell death (E), migration (F), as well as invasion (G) were examined, respectively. **(H)** Effect of chemical inhibition of CYP1B1 on CASP1 activation. CASP1 activity was determined by colorimetric assay in PC-3 cells treated with the indicated concentration of TMS. **(I)** Effect of CASP1 inhibition on apoptotic cell death induced by chemical inhibition of CYP1B1. Apoptotic cell death was determined by flow cytometric analysis using double staining with Annexin V-FITC and 7-AAD in TMS-treated PC-3 cells with or without addition of Z-YVAD-fmk (100 μM). **(J and K)** Effect of CYP1B1 activation on CASP1 in RWPE-1 cells. CASP1 expression was determined by Western blot after CYP1B1 overexpression (J). CASP1 activity was examined by colorimetric assay in cells treated with the indicated concentration of DMBA (K). *p<0.05; **p<0.01; ***p<0.001.

To delineate the mechanism of the CYP1B1 inhibition-induced antitumor effect, we looked for changes in gene expression in PC-3/CYP1B1 shRNA #4-2 cells using the Human Apoptosis RT^2^ Profiler PCR Array and Human CancerPathFinder RT^2^ Profiler PCR Array. From the genes analyzed with both arrays, we observed that the expression of 6 genes was significantly increased while the expression of 6 other genes was decreased (Table 1). To verify the array data, we performed qPCR assay using probes with a different sequence from the one used in the PCR arrays in both PC-3/CYP1B1 shRNA #4-2 and #4-3 cells. Among the genes identified from the PCR Array assays, changes in tumor necrosis factor receptor superfamily, member 9 (TNFRSF9), caspase-1 (CASP1), lymphotoxin alpha (LTA), CD27, spleen tyrosine kinase (SYK), FBJ murine osteosarcoma viral oncogene homolog (FOS), interleukin 8 (IL8), thrombospondin 1 (THBS1), and V-myc myelocytomatosis viral oncogene homolog (MYC) were confirmed and further analyzed (Supplementary Figure 3A and Figure 4A). As shown by Western blotting, only TNFRSF9, CASP1, and THBS1 mRNA levels were associated with changes in protein expression (Supplementary Figure 3B and 4B). TNFRSF9 and THBS1 were the most dramatically up- and down-regulated genes respectively among those examined and CASP1 also was induced 4~6-fold over controls. Among these, CASP1 has been shown to be significantly down-regulated in prostate cancer [15, 16] and its genetic restoration reduces the tumorigenic potential by increasing apoptotic sensitivity [27]. Thus, we hypothesize CASP1 to be a potential target of CYP1B1-mediated tumorigenic activity and antitumor effect of CYP1B1 inhibition.

**Table 1:** Summary of genes significantly altered by CYP1B1 inhibition

Symbol	Fold Change	Description	Array
TNFRSF9	4.6	Tumor necrosis factor receptor superfamily, member 9	Apoptosis
CASP1	4.1	Caspase 1, apoptosis-related cysteine peptidase (interleukin 1, beta, convertase)	Apoptosis
CD27	3.5	CD27 molecule	Apoptosis
LTA	3.4	Lymphotoxin alpha (TNF superfamily, member 1)	Apoptosis
BIRC8	3.0	Baculoviral IAP repeat containing 8	Apoptosis
HRK	2.9	Harakiri, BCL2 interacting protein(contains only BH3 domain)	Apoptosis
SYK	0.01	Spleen tyrosine kinase	Cancer PathFinder
FOS	0.08	FBJ murine osteosarcoma viral oncogene homolog	Cancer PathFinder
IL8	0.11	Interleukin 8	Cancer PathFinder
THBS1	0.12	Thrombospondin 1	Cancer PathFinder
MYC	0.37	V-myc myelocytomatosis viral oncogene homolog	Cancer PathFinder
NFKBIA	0.46	Nuclear factor of kappa light polypeptide gene enhancer in B-cells inhibitor, alpha	Cancer PathFinder

### CYP1B1 inhibition activates CASP1-dependent anti-tumorigenic activity

In addition to PC-3 cells, CYP1B1 knockdown induces CASP1 mRNA and protein expression in DU145 cells (Figure 4A and 4B). Consistent with upregulation at the gene level, CASP1 enzyme activity was also significantly enhanced in both CYP1B1 knockdown cells (Figure 4C). To determine the impact of CASP1 on the CYP1B1 inhibition-mediated anti-tumor effect, we examined the rate of cell proliferation and apoptosis. Treatment with Z-YVAD-fmk, a specific inhibitor of CASP1, accelerated the rates of proliferation in CYP1B1 knockdown cells (Figure 4D). This may be due to the reduction of apoptosis by CASP1 inhibition (Figure 4E). Next, we evaluated the effect of CASP1 in the low migration and invasiveness caused by CYP1B1 inhibition. As shown in Figure 4F and 4G, treatment with Z-YVAD-fmk restored cell migratory and invasive characteristics of PCa cells, respectively. In addition to the genetic approach, CASP1 activity was increased by TMS, a chemical inhibitor of CYP1B1 (Figure 4H). Incubation with CASP1 inhibitor deceased TMS-induced apoptosis of PC-3 cells (Figure 4I). On the other hand, CYP1B1 overexpression or activation by DMBA reduced expression and activity of CASP1 in RWPE-1 cells (Figure 4J and 4K). These results indicate that CASP1 plays a critical role in CYP1B1-mediated tumorigenicity in PCa.

### Up-regulation of CYP1B1 is associated with clinicopathologic characteristics of PCa patients

**Figure 5:**
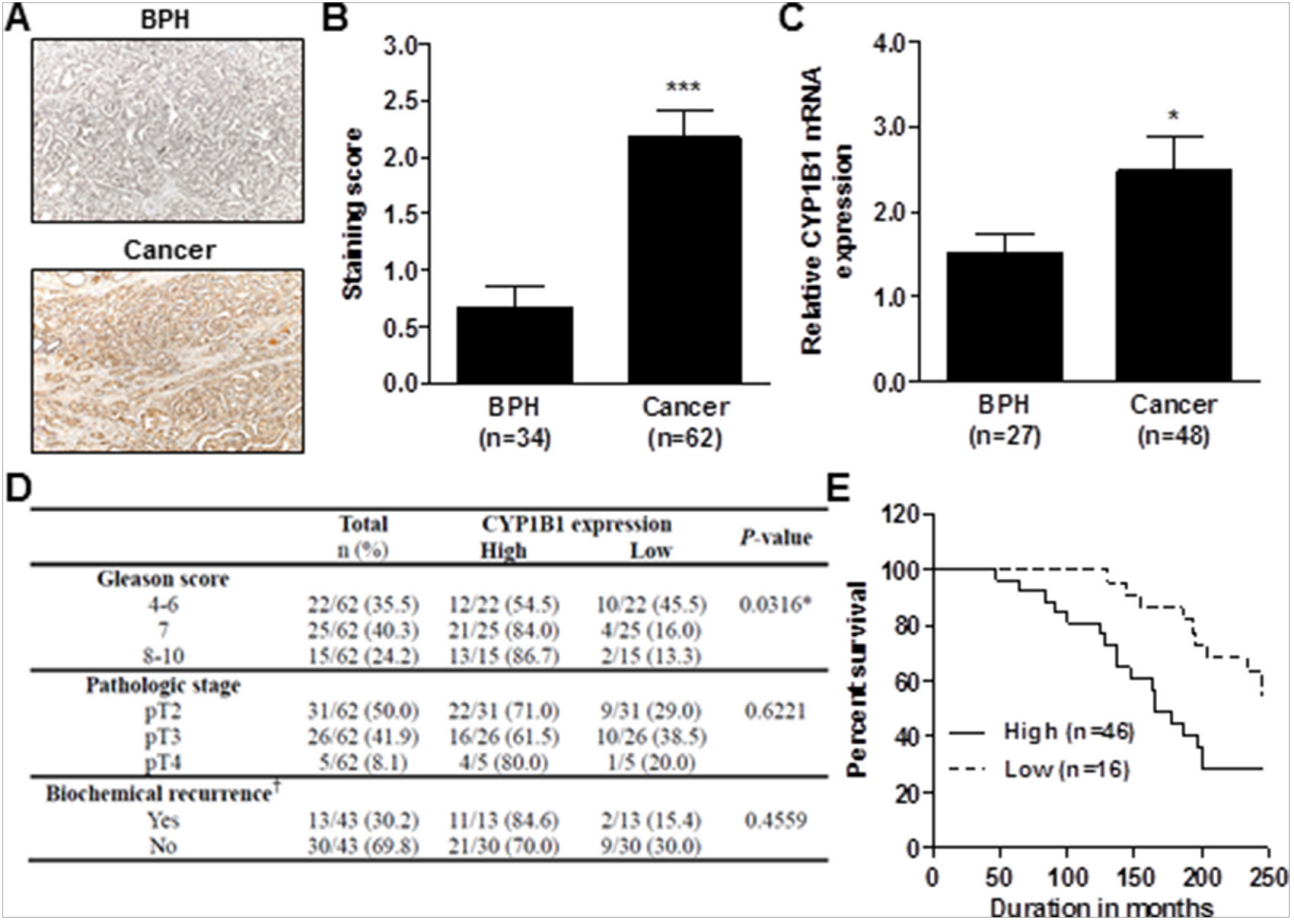
Association of CYP1B1 expression with clinicopathologic characteristics of PCa **(A–C)** CYP1B1 expression in PCa tissues. Immunohistochemical staining of CYP1B1 protein in PCa specimens. Representative images showing immunoreactive CYP1B1 in BPH and cancer tissues (magnification: ×200) (A). Summary of CYP1B1 immunostaining score. Staining intensity was assessed as described in Materials and Methods (B). CYP1B1 mRNA expression in microdissected prostate tissues (C). **(D)** Correlation of CYP1B1 expression with clinicopathological characteristics of patients with PCa. †Data was not available for some samples. *p<0.05; ***p<0.001 **(E)** Kaplan-Meier survival curves for overall survival of patients with PCa. *p*=0.0111.

As shown in Figure 5A, most CYP1B1 protein was expressed in the glandular epithelium of PCa tissues. At the cellular level, it was detected in both the nucleus and cytoplasm. In contrast, weak or no epithelial CYP1B1 expression was found in benign prostatic hypertrophy (BPH) tissues with average staining scores of 0.67±0.20 (versus 2.17±0.22 in cancer tissues) (Figure 5B). The level of CYP1B1 mRNA was also significantly increased in PCa tissues (Figure 5C). Although high expression of CYP1B1 has been demonstrated in prostate tumors, there are no reports regarding the correlation of its expression with clinicopathologic characteristics. Thus, we further analyzed the expression pattern of CYP1B1 in clinical tissues to examine its relationship with clinicopathologic characteristics such as Gleason score, pathologic stage, and biochemical recurrence of PCa. Clinical demographics of the study cohort are summarized in Supplementary Table 1. Increased CYP1B1 expression (staining score ≥ 2.0) was observed in 54.5% of patients with low Gleason score (4-6), 84.0% of patients with Gleason score 7, and 86.7% of patients with higher Gleason score (8-10) (Figure 5D). This result suggests that CYP1B1 expression tends to increase in higher grades of PCa. No statistical significance was observed between CYP1B1 expression and pathologic stage but up-regulation of CYP1B1 was detected in 71.0% of pT2 patients, 61.5% of pT3 patients, and 80% of pT4 patients. Similarly, CYP1B1 was also augmented in 11 of 13 cases (84.6%) with biochemical recurrence in the patient samples (Figure 5D). Furthermore, overall survival was significantly reduced in patients with high levels of CYP1B1 protein (Figure 5E).

### CYP1B1 expression is associated with CASP1 levels in PCa tissue

**Figure 6:**
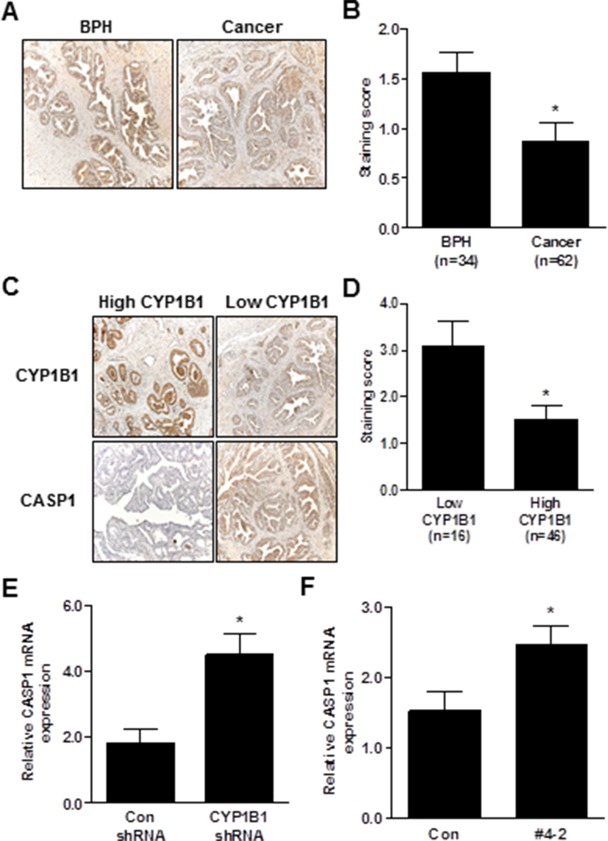
Inverse correlation between CYP1B1 and CASP1 expression **(A and B)** Immunohistochemical staining of CASP1 protein in PCa specimens. Representative images showing immunoreactive caspase-1 in BPH and cancer tissues (magnification: × 200) (A). Summary of CASP1 immunostaining score (B). **(C and D)** Immunohistochemical staining of CYP1B1 and CASP1 protein in prostate cancer specimens. Representative images showing inverse correlation of CYP1B1 and CASP1 in BPH and cancer tissues (magnification: × 200) (C). Summary of CASP1 immunostaining score (D). **(E and F)** Expression of CASP1 mRNA in tumors injected with CYP1B1 shRNA (E) and PC-3/CYP1B1 shRNA #4-2 xenografts (F). *p<0.05.

To determine whether CYP1B1 expression is correlated with CASP1 level, we first examined the level of CASP1 in prostate cancer tissue samples. As shown in Figure 6A and 6B, CASP1 expression was significantly down-regulated in human PCa. However, there was no statistically significant correlation between CASP1 expression and clinicopathological characteristics (data not shown). Next, we categorized tissue specimens as either high or low CYP1B1 expression groups as previously determined (Figure 5D) and measured CASP1 expression. As shown in Figure 6C and 6D, prostate tumors with strong CYP1B1 expression showed low CASP1 levels. On the contrary, PCa samples with relatively low CYP1B1 expression had a significantly higher level of CASP1 expression as compared to those with high CYP1B1 expression. In addition, increased CASP1 expression was found in the tumors injected with or stably expressing CYP1B1 shRNA (Figure 6E and 6F).

## DISCUSSION

Since CYP1B1 is implicated as an important factor in the development of various cancers, understanding the precise mechanisms of CYP1B1-mediated cancer progression is required in the development of new strategies for cancer treatment. CYP1B1 provokes an oncogenic phenotype by damaging DNA through the formation of 8-hydroxy-2′-deoxyguanosine [28]. Recently, several molecular targets of CYP1B1 have been found and indicate the participation of CYP1B1 in multiple pathways during the progression of various types of cancer [3, 4, 6, 7]. In line with these efforts, our study found that CASP1 is a critical mediator of CYP1B1-induced tumorigenicity in PCa.

CASP1 is an apical activator of the cell death pathway [9] and its overexpression has been shown to induce apoptosis in mammalian cells [12, 29]. Exogenous expression of CASP1 markedly reduced the growth of renal cancer cells *in vitro* and *in vivo*, and silencing CASP1 activity resulted in the establishment of solid tumors [30]. Down-regulation of CASP1 was found in human colon cancer [31] and enhanced tumor formation in a colitis-associated colorectal cancer model [13]. Furthermore, CASP1 is frequently downregulated in prostate cancer [15, 16] and its genetic restoration reduces the tumorigenic potential via apoptosis [27, 32]. 2,3,7,8-Tetrachlorodibenzo-p-dioxin, a strong CYP1B1 activator, suppressed activities of CASP1 and CASP3 in the apoptotic cell death of hepatocytes [33]. In this study, we found that CASP1 is significantly up-regulated by CYP1B1 inhibition and is a newly identified target molecule of CYP1B1-mediated tumorigenesis. CASP1 is silenced by DNA methylation in renal cancer [30]. Given that CYP1B1 knockdown increases CASP1 (Figure 4), we suggest an alternative mechanism for CASP1 suppression and its critical role in CYP1B1-mediated regulation of PCa progression.

According to Gregoraszczuk *et al*., 17β-estradiol (E_2_) increases CYP1B1 protein expression, changing the local metabolic activation pathway to increased 4-OHE_2_ production. Both E_2_ and 4-OHE_2_ decrease CASP9 activity [34]. Therefore, it is possible that reduction of 4-OHE_2_ levels by CYP1B1 suppression may lead to CASP1 activation. CYP enzyme promotes reactive oxygen species (ROS) generation which occurs during the metabolic conversion of procarcinogens to their ultimate reactive electrophilic intermediates [35]. However, interestingly, a lack of CYP1B1 expression and/or activity leads to accumulation of ROS and increased intracellular oxidative stress in retinal endothelial cells, perivascular supporting cells, and trabecular meshwork cells [36–38]. Moreover, CASP1 can be activated by ROS [39, 40]. Therefore, it is plausible that ROS produced by CYP1B1 inhibition activates CASP1 which induces antitumor effects in PCa cells. Due to a contradictory report suggesting that CYP1B1 shRNA reduces ROS production in vascular smooth muscle cell [41], this hypothesis needs further clarification. In contrast to previous findings [27, 30–32], CASP1 has an anti-apoptotic effect in pancreatic carcinoma [42]. Thus, it would be of interest to examine the expression and function of CYP1B1 in pancreatic cancer and determine its relationship with CASP1.

To analyze the therapeutic potential of lentivirus-delivered CYP1B1 shRNA, we performed a pre-clinical study. The recombinant lentivirus expressing CYP1B1 shRNA was intratumorally injected into established tumors or tumor cells expressing CYP1B1 shRNA were injected into nude mice. Both approaches suppressed the growth of tumors arising from PC-3 cells. These results demonstrate that CYP1B1 inhibition by lentivirus-mediated shRNA resulted in a beneficial effect *in vivo*. To our knowledge, our study is the first report determining the therapeutic effect of lentivirus expressing CYP1B1 shRNA. Shariat *et al*. showed that an adenoviral vector expressing inducible form of CASP1 can inhibit the growth of PCa *in vitro* and *in vivo* by the induction of apoptosis [32]. In contrast to adenoviruses, a lentivirus-based strategy does not trigger a potentially dangerous immune response. Therefore, it is ideally suited for use in human cancer cells. Further development of a lentiviral vector expressing CYP1B1 shRNA may result in a protective effect *in vivo* against PCa.

CYP1B1 is overexpressed in a variety of human tumor cells including prostate cancer [2, 22, 43, 44]. However, its association with clinicopathological features has not been elucidated. Here, we demonstrate that high levels of CYP1B1 are associated with parameters such as Gleason score and survival rate of PCa patients. Thus, CYP1B1 could be a potential prognostic and diagnostic marker for PCa. We also found that CYP1B1 expression is inversely associated with CASP1 levels in human PCa tissues (Figure 6). Consistent with a previous study [15], we could find no statistically significant correlation between CASP1 expression and clinicopathological parameters including Gleason score of PCa tissues probably due to the relatively small number of tissue samples. Analysis of a larger group of tumors with more cases in the different histological grade categories will be required to determine an association between CASP1 expression and clinical parameters of PCa. In addition, it will be of interest to perform studies on the association of CYP1B1 expression and its polymorphic variants with CASP1 expression in PCa patients.

In summary, the present study demonstrates that CYP1B1 induces tumorigenecity of PCa cells by modulating CASP1 expression. We also presented data indicating the feasibility of using *in vivo* lentivirus-delivered CYP1B1 shRNA to reduce the tumor burden in animals. This result supports the idea that attenuation of CYP1B1 may be useful in the treatment of PCa. Finally, we show the clinical relevance and inverse correlation between CYP1B1 and CASP1 in human tissue specimens. Thus, we believe the findings of this study may provide a new mechanistic interpretation for CYP1B1-induced development and progression of PCa.

## MATERIALS AND METHODS

### Cell lines and reagents

Human PCa cell lines (LNCaP, PC-3, and DU145) and a non-malignant epithelial prostate cell line (RWPE-1) were purchased from the ATCC (Manassas, VA). Keratinocyte serum-free medium (K-SFM), bovine pituitary extract and human recombinant epidermal growth factor were purchased from Invitrogen (Carlsbad, CA). RPMI 1640, EMEM, Opti-MEM and penicillin/streptomycin mixtures were obtained from the UCSF Cell Culture Facility (San Francisco, CA). Fetal bovine serum (FBS) was a product of Atlanta Biologicals (Lawrenceville, GA). Z-YVAD-fmk was purchased from Santa Cruz Biotechnology (Santa Cruz). N-acetylcysteine (NAC), 7,12-Dimethylbenz[a]anthracene (DMBA), and 2,3′,4,5′-Tetramethoxystilbene (TMS) were obtained from Sigma (St. Louis, MO).

### Cell culture

LNCaP and PC-3 cells were grown in RPMI 1640 and DU145 cells were cultured in EMEM. All culture medium contained 10% FBS and 100 μg/ml penicillin/streptomycin. RWPE-1 cells were cultured in K-SFM supplemented with 0.05 mg/ml bovine pituitary extract and 5 ng/ml human recombinant epidermal growth factor. All cell lines were maintained at 37°C in a humidified atmosphere composed of 5% CO_2_ and 95% air.

### Western blot analysis

Whole cell extracts from cultured cells were prepared using radioimmunoprecipitation assay buffer (Thermo Scientific, Waltham, MA) containing protease inhibitor cocktail (Roche Diagnostics, Risch-Rotkreuz, Swizerland). Immunoblotting was carried out according to standard protocols with antibodies against TNFRSF9 (Sigma), LTA (GeneTex, San Antonio, TX), CYP1B1, CD27, IL8, THBS1 (Abcam, Cambridge, MA), SYK, FOS, MYC, NFKBIA, CASPASE-1 (Cell Signaling Technology, Danvers, MA). Antibody against GAPDH (Santa Cruz Biotechnology, Santa Cruz, CA) and β-actin (Abcam) was used to confirm equal loading.

### Establishment of stably expressing plasmid or short hairpin (sh) RNA specific for CYP1B1

Ectopic expression of CYP1B1 plasmid (OriGene Techologies, Rockville, MD) in RWPE-1 cells and CYP1B1 shRNA (Origene) in PC-3 and DU145 cells along with their controls were achieved using lentivirus as per the manufacturer's instructions. The transduced cells were then selected using puromycin (1 μg/ml; Sigma).

### Cell proliferation and colony formation assay

For 3-(4,5-dimethylthiazol-2-yl)-5-(3-carboxymethoxyphenyl)-2-(4-sulfophenyl)-2H-tetrazolium (MTS)-based cell proliferation assays, cells were plated in triplicate in 96-well plates at a density of 5×10^3^ cells per well. At the desired time point, the number of viable cells was determined by adding CellTiter 96 AQueous One Solution reagent (Promega, Madison, WI) to each well and measuring the absorbance at 490 nm on a SpectraMax 190 plate reader (Molecular Devices, Sunnyvale, CA). Results were expressed as percent optical density with absorbance of control cells being 100%. To assess colony formation, cells were plated in triplicate in 6-well plates at a density of 1×10^2^ cells per well. At the desired time point, colonies were identified by adding 1% paraformaldehyde followed by incubating in 0.1% crystal violet. The number of colonies was quantified by dissolving them in methanol and measuring the absorbance at 540 nm on a SpectraMax 190 plate reader.

### Apoptosis and cell cycle assay

For apoptosis assays, cells were stained with an Annexin V-fluorescein isothiocyanate (FITC)/7-amino-actinomycin D (7-AAD) (BD Biosciences, San Diego, CA) as described by the manufacturer. Both early and late apoptotic cells were included in cell death determinations. For cell cycle analysis, cells were stained with 4′, 6-diamidino-2-phenylindole (DAPI). Both assays were analyzed by a Cell Lab Quanta™ SC MPL (Beckman Coulter, Fullerton, CA).

### Migration and invasion assay

Cell migration and invasion were measured using the CytoSelectTM 24-Well Cell Migration and Invasion Assay (Cell Biolabs, San Diego, CA). Stained invasive and migratory cells were observed with a Nikon microscope and quantified using a SpectraMax plate reader at 560 nm.

### *In vivo* intratumoral delivery and xenograft mouse model

All animal care was in accordance with current guidelines and this study was approved by the San Francisco Veterans Affairs IACUC (Institutional Animal Care and Use Committee). For intratumoral injection of lentivirus-mediated CYP1B1 shRNA, five week old male mice (Charles River, Burlington, MA) were injected subcutaneously into the dorsal flank area with 200 μl of RPMI 1640 medium containing PC-3 cells (5×10^6^ cells). Once tumors reached a volume of 30-40 mm^3^, the tumors were injected every 3 days with 50 μl (4×10^7^ cells) of viral particles containing CYP1B1 or control shRNA. For the subcutaneous xenograft mouse model, PC-3 cells (5×10^6^ cells) stably transfected with either CYP1B1 shRNA #4-2 or control shRNA suspended in 100 μl RPMI 1640 medium were subcutaneously injected into the right backside flank of five week old male nude mice. Six to eight nude mice were used per treatment group and tumor growth was examined over the course of 35 days. Tumor volume was calculated on the basis of width (x) and length (y) using the formula: x^2^y/2, where x<y.

### Immunohistochemistry

Written informed consent for the use of the tissues was obtained from all patients before surgery, and the study was approved by the Clinical Research Office of the San Francisco Veterans Affairs Medical Center and the Institutional Review Board of the University of California at San Francisco. Immunostaining was performed on formalin-fixed paraffin embedded PCa sections using the VECTASTAIN ABC Kit (Vector Laboratories, Burlingame, CA) according to the manufacturer's instructions. After incubation with anti-CYP1B1 and CASP1 antibodies (Abcam), ImmPACT DAB (Vector Laboratories) was added as chromogen followed by counterstaining with hematoxylin. Staining intensity of each tissue section was visually evaluated with an Olympus BX60 microscope equipped with Spot Advanced software (Diagnostic Instruments, Sterling Heights, MI) and was ranked on an overall scale from 0 to 3; with 0 indicating the absence of staining; 1, weak staining; 2, moderate staining; and 3, strong staining. For Ki67 staining, tumor sections were stained with Anti-Ki67 antibody (Abcam) and positive cells were counted from at least 3 randomly selected microscopic fields with % positivity calculated.

### Quantitative RT-PCR

Total RNA was isolated using the RNeasy Mini Kit (Qiagen, Valencia, CA, USA) and was converted into cDNA by using the iScript™ cDNA Synthesis Kit (Bio-Rad, Hercules, CA) according to the manufacturer's instructions. To assess gene expression, cDNAs were amplified with the TaqMan® Gene Expression Assays and TaqMan Fast Universal PCR Master Mix using the 7500 Fast Real-Time PCR System (Applied Biosystems, Foster City, CA). To investigate the expression of genes regulated by CYP1B1 inhibition, the RT^2^ Profiler PCR Array Human Apoptosis and Human Cancer PathwayFinder (SABiosciences, Frederick, MD) were used as per manufacturer's instructions. Each array contains quantitative PCR primers of 84 known apoptosis- or tumorigenesis-related genes, respectively. The relative change in gene expression was calculated by the comparative Ct (threshold cycle) method using the 7500 Fast System Sequence Detection Software (Applied Biosystems).

### Caspase-1 enzymatic activity assay

Caspase-1 activation was measured using a Caspase-1/ICE Colorimetric Assay Kit as described by the supplier's instructions (R&D Systems, Minneapolis, MN).

### Statistical analysis

Values in Figures are presented as the mean±standard error of mean (SEM) based on results obtained from at least three independent experiments. For *in vivo* studies, results are based on six animals per group. All statistical analyses were carried out using GraphPad PRISM Software. Two-tailed unpaired Student's t-test was used for comparisons between two groups. Chi-square test was used for analyzing the correlation between clinicopathologic parameters and CYP1B1 protein expression. Significance of percent survival was done with log-rank test. A P value of <0.05 was regarded as statistically significant.

## SUPPLEMENTARY MATERIALS FIGURES AND TABLES



## Abbreviations

CYP1B1: Cytochrome P450 1B1
CASP1: caspase-1
PCa: prostate cancer
RCC: renal cell carcinoma
IL: interleukin
K-SFM: Keratinocyte serum-free medium
FBS: Fetal bovine serum
NAC: N-acetylcysteine
DMBA: 7,12-Dimethylbenz[a]anthracene
TMS: 2,3′,4,5′-Tetramethoxystilbene
shRNA: Short hairpin RNA
MTS: 3-(4,5-dimethylthiazol-2-yl)-5-(3-carboxymethoxyphenyl)-2-(4-sulfophenyl)-2H-tetrazolium
FITC: fluorescein isothiocyanate
7-AAD: 7-amino-actinomycin D
DAPI: 4′, 6-diamidino-2-phenylindole
qRT-PCR: quantitative reverse transcription polymerase chain reaction
ROS: reactive oxygen species
TNFRSF9: tumor necrosis factor receptor superfamily, member 9
LTA: lymphotoxin alpha
SYK: spleen tyrosine kinase
FOS: FBJ murine osteosarcoma viral oncogene homolog
IL8: interleukin 8
THBS1: thrombospondin 1
MYC: V-myc myelocytomatosis viral oncogene homolog
E_2_: 17β-estradiol

## References

[1] Murray GI (2000). The role of cytochrome P450 in tumour development and progression and its potential in therapy. J Pathol.

[2] Murray GI, Taylor MC, McFadyen MC, McKay JA, Greenlee WF, Burke MD, Melvin WT (1997). Tumor-specific expression of cytochrome P450 CYP1B1. Cancer Res.

[3] Saini S, Hirata H, Majid S, Dahiya R (2009). Functional significance of cytochrome P450 1B1 in endometrial carcinogenesis. Cancer Res.

[4] Shatalova EG, Klein-Szanto AJ, Devarajan K, Cukierman E, Clapper ML (2011). Estrogen and cytochrome P450 1B1 contribute to both early- and late-stage head and neck carcinogenesis. Cancer Prev Res (Phila).

[5] Chang I, Mitsui Y, Fukuhara S, Gill A, Wong DK, Yamamura S, Shahryari V, Tabatabai ZL, Dahiya R, Shin DM, Tanaka Y (2015). Loss of miR-200c up-regulates CYP1B1 and confers docetaxel resistance in renal cell carcinoma. Oncotarget.

[6] Mitsui Y, Chang I, Fukuhara S, Hiraki M, Arichi N, Yasumoto H, Hirata H, Yamamura S, Shahryari V, Deng G, Wong DK, Majid S, Shiina H (2015). CYP1B1 promotes tumorigenesis via altered expression of CDC20 and DAPK1 genes in renal cell carcinoma. BMC Cancer.

[7] Kwon YJ, Baek HS, Ye DJ, Shin S, Kim D, Chun YJ (2016). CYP1B1 Enhances Cell Proliferation and Metastasis through Induction of EMT and Activation of Wnt/beta-Catenin Signaling via Sp1 Upregulation. PLoS One.

[8] Thornberry NA, Bull HG, Calaycay JR, Chapman KT, Howard AD, Kostura MJ, Miller DK, Molineaux SM, Weidner JR, Aunins J (1992). A novel heterodimeric cysteine protease is required for interleukin-1 beta processing in monocytes. Nature.

[9] Zhang WH, Wang X, Narayanan M, Zhang Y, Huo C, Reed JC, Friedlander RM (2003). Fundamental role of the Rip2/caspase-1 pathway in hypoxia and ischemia-induced neuronal cell death. Proc Natl Acad Sci U S A.

[10] Kang SJ, Wang S, Hara H, Peterson EP, Namura S, Amin-Hanjani S, Huang Z, Srinivasan A, Tomaselli KJ, Thornberry NA, Moskowitz MA, Yuan J (2000). Dual role of caspase-11 in mediating activation of caspase-1 and caspase-3 under pathological conditions. J Cell Biol.

[11] Shibata M, Hisahara S, Hara H, Yamawaki T, Fukuuchi Y, Yuan J, Okano H, Miura M (2000). Caspases determine the vulnerability of oligodendrocytes in the ischemic brain. J Clin Invest.

[12] Miura M, Zhu H, Rotello R, Hartwieg EA, Yuan J (1993). Induction of apoptosis in fibroblasts by IL-1 beta-converting enzyme, a mammalian homolog of the C. elegans cell death gene ced-3. Cell.

[13] Hu B, Elinav E, Huber S, Booth CJ, Strowig T, Jin C, Eisenbarth SC, Flavell RA (2010). Inflammation-induced tumorigenesis in the colon is regulated by caspase-1 and NLRC4. Proc Natl Acad Sci U S A.

[14] Denes A, Lopez-Castejon G, Brough D (2012). Caspase-1: is IL-1 just the tip of the ICEberg?. Cell Death Dis.

[15] Winter RN, Kramer A, Borkowski A, Kyprianou N (2001). Loss of caspase-1 and caspase-3 protein expression in human prostate cancer. Cancer Res.

[16] Ummanni R, Lehnigk U, Zimmermann U, Woenckhaus C, Walther R, Giebel J (2010). Immunohistochemical expression of caspase-1 and -9, uncleaved caspase-3 and -6, cleaved caspase-3 and -6 as well as Bcl-2 in benign epithelium and cancer of the prostate. Exp Ther Med.

[17] Guo Y, Kyprianou N (1999). Restoration of transforming growth factor beta signaling pathway in human prostate cancer cells suppresses tumorigenicity via induction of caspase-1-mediated apoptosis. Cancer Res.

[18] Siegel RL, Miller KD, Jemal A (2016). Cancer statistics, 2016. CA Cancer J Clin.

[19] Albertsen PC, Hanley JA, Fine J (2005). 20-year outcomes following conservative management of clinically localized prostate cancer. JAMA.

[20] Mimeault M, Batra SK (2006). Recent advances on multiple tumorigenic cascades involved in prostatic cancer progression and targeting therapies. Carcinogenesis.

[21] Carnell DM, Smith RE, Daley FM, Barber PR, Hoskin PJ, Wilson GD, Murray GI, Everett SA (2004). Target validation of cytochrome P450 CYP1B1 in prostate carcinoma with protein expression in associated hyperplastic and premalignant tissue. Int J Radiat Oncol Biol Phys.

[22] Tokizane T, Shiina H, Igawa M, Enokida H, Urakami S, Kawakami T, Ogishima T, Okino ST, Li LC, Tanaka Y, Nonomura N, Okuyama A, Dahiya R (2005). Cytochrome P450 1B1 is overexpressed and regulated by hypomethylation in prostate cancer. Clin Cancer Res.

[23] Cavalieri EL, Devanesan P, Bosland MC, Badawi AF, Rogan EG (2002). Catechol estrogen metabolites and conjugates in different regions of the prostate of Noble rats treated with 4-hydroxyestradiol: implications for estrogen-induced initiation of prostate cancer. Carcinogenesis.

[24] Williams JA, Martin FL, Muir GH, Hewer A, Grover PL, Phillips DH (2000). Metabolic activation of carcinogens and expression of various cytochromes P450 in human prostate tissue. Carcinogenesis.

[25] Kobayashi M, Ishida H, Shindo T, Niwa S, Kino M, Kawamura K, Kamiya N, Imamoto T, Suzuki H, Hirokawa Y, Shiraishi T, Tanizawa T, Nakatani Y (2008). Molecular analysis of multifocal prostate cancer by comparative genomic hybridization. Prostate.

[26] Gajjar K, Martin-Hirsch PL, Martin FL (2012). CYP1B1 and hormone-induced cancer. Cancer Lett.

[27] Winter RN, Rhee JG, Kyprianou N (2004). Caspase-1 enhances the apoptotic response of prostate cancer cells to ionizing radiation. Anticancer Res.

[28] Klaunig JE, Wang Z, Pu X, Zhou S (2011). Oxidative stress and oxidative damage in chemical carcinogenesis. Toxicol Appl Pharmacol.

[29] Alnemri ES, Fernandes-Alnemri T, Litwack G (1995). Cloning and expression of four novel isoforms of human interleukin-1 beta converting enzyme with different apoptotic activities. J Biol Chem.

[30] Ueki T, Takeuchi T, Nishimatsu H, Kajiwara T, Moriyama N, Narita Y, Kawabe K, Ueki K, Kitamura T (2001). Silencing of the caspase-1 gene occurs in murine and human renal cancer cells and causes solid tumor growth in vivo. Int J Cancer.

[31] Jarry A, Vallette G, Cassagnau E, Moreau A, Bou-Hanna C, Lemarre P, Letessier E, Le Neel JC, Galmiche JP, Laboisse CL (1999). Interleukin 1 and interleukin 1beta converting enzyme (caspase 1) expression in the human colonic epithelial barrier. Caspase 1 downregulation in colon cancer. Gut.

[32] Shariat SF, Desai S, Song W, Khan T, Zhao J, Nguyen C, Foster BA, Greenberg N, Spencer DM, Slawin KM (2001). Adenovirus-mediated transfer of inducible caspases: a novel “death switch” gene therapeutic approach to prostate cancer. Cancer Res.

[33] Patterson RM, Stachlewitz R, Germolec D (2003). Induction of apoptosis by 2,3,7,8-tetrachlorodibenzo-p-dioxin following endotoxin exposure. Toxicol Appl Pharmacol.

[34] Gregoraszczuk E, Ptak A (2011). Involvement of caspase-9 but not caspase-8 in the anti-apoptotic effects of estradiol and 4-OH-Estradiol in MCF-7 human breast cancer cells. Endocr Regul.

[35] Valko M, Leibfritz D, Moncol J, Cronin MT, Mazur M, Telser J (2007). Free radicals and antioxidants in normal physiological functions and human disease. Int J Biochem Cell Biol.

[36] Palenski TL, Sorenson CM, Jefcoate CR, Sheibani N (2013). Lack of Cyp1b1 promotes the proliferative and migratory phenotype of perivascular supporting cells. Lab Invest.

[37] Tang Y, Scheef EA, Wang S, Sorenson CM, Marcus CB, Jefcoate CR, Sheibani N (2009). CYP1B1 expression promotes the proangiogenic phenotype of endothelium through decreased intracellular oxidative stress and thrombospondin-2 expression. Blood.

[38] Zhao Y, Wang S, Sorenson CM, Teixeira L, Dubielzig RR, Peters DM, Conway SJ, Jefcoate CR, Sheibani N (2013). Cyp1b1 mediates periostin regulation of trabecular meshwork development by suppression of oxidative stress. Mol Cell Biol.

[39] Dostert C, Petrilli V, Van Bruggen R, Steele C, Mossman BT, Tschopp J (2008). Innate immune activation through Nalp3 inflammasome sensing of asbestos and silica. Science.

[40] Harijith A, Ebenezer DL, Natarajan V (2014). Reactive oxygen species at the crossroads of inflammasome and inflammation. Front Physiol.

[41] Yaghini FA, Song CY, Lavrentyev EN, Ghafoor HU, Fang XR, Estes AM, Campbell WB, Malik KU (2010). Angiotensin II-induced vascular smooth muscle cell migration and growth are mediated by cytochrome P450 1B1-dependent superoxide generation. Hypertension.

[42] Schlosser S, Gansauge F, Ramadani M, Beger HG, Gansauge S (2001). Inhibition of caspase-1 induces cell death in pancreatic carcinoma cells and potentially modulates expression levels of bcl-2 family proteins. FEBS Lett.

[43] McFadyen MC, Cruickshank ME, Miller ID, McLeod HL, Melvin WT, Haites NE, Parkin D, Murray GI (2001). Cytochrome P450 CYP1B1 over-expression in primary and metastatic ovarian cancer. Br J Cancer.

[44] McFadyen MC, Breeman S, Payne S, Stirk C, Miller ID, Melvin WT, Murray GI (1999). Immunohistochemical localization of cytochrome P450 CYP1B1 in breast cancer with monoclonal antibodies specific for CYP1B1. J Histochem Cytochem.

