# Correction: Cytochrome P450 1B1 inhibition suppresses tumorigenicity of prostate cancer *via* caspase-1 activation

**DOI:** 10.18632/oncotarget.26197

**Published:** 2018-09-25

**Authors:** Inik Chang, Yozo Mitsui, Seul Ki Kim, Ji Su Sun, Hye Sook Jeon, Jung Yun Kang, Nam Ju Kang, Shinichiro Fukuhara, Ankurpreet Gill, Varahram Shahryari, Z. Laura Tabatabai, Kirsten L. Greene, Rajvir Dahiya, Dong Min Shin, Yuichiro Tanaka

**Affiliations:** ^1^ Department of Oral Biology, Yonsei University College of Dentistry, Seoul, South Korea; ^2^ Department of Surgery and Division of Urology, Veterans Affairs Medical Center, San Francisco, California, United States of America; ^3^ Department of Urology, University of California, San Francisco, California, United States of America; ^4^ BK21 PLUS Project, Yonsei University College of Dentistry, Seoul, South Korea; ^5^ Department of Pathology, Veterans Affairs Medical Center and University of California, San Francisco, California, United States of America

**This article has been corrected:** The image for #4-3 in Figure 2C is incorrect. It is a duplicate image of panel #4-2. The corrected Figure 2C is shown below. The authors declare that these corrections do not change the results or conclusions of this paper.

**Figure 2:**
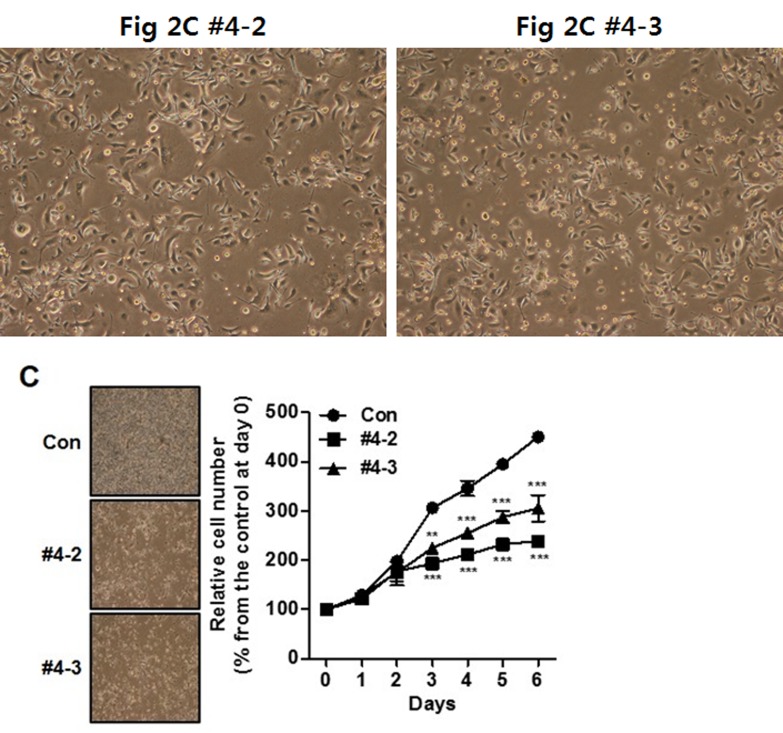
CYP1B1 inhibition suppresses *in vitro* tumorigenicity (**A** and **B**) Expression of CYP1B1 mRNA (A) and protein (B) in CYP1B1 shRNA or control shRNA expressing PC-3 cells. Levels were determined by qRT-PCR and Western blot, respectively. (**C**–**H**) Effect of CYP1B1 knockdown on *in vitro* tumorigenicity. Cell proliferation as determined by MTS assay at the indicated times. Representative images of cell morphology (left panel) and quantification of cell proliferation (right panel) are shown (C). Colony formation as determined by crystal violet staining. Representative image of colonies (left panel) and quantification of stained colonies (right panel) are shown (D). Apoptotic cell death as determined by flow cytometric analysis using double staining with Annexin V-FITC and 7-AAD. Representative biparametric histograms exhibiting cell (left panel) and quantification of apoptotic cells (right panel) are shown (E). Cell cycle progression as determined by DAPI staining (F). Cell migration (G) and invasion (H) capability as determined by transwell migration and invasion assay, respectively. Representative images (left panel) and quantification of assay (right panel) are shown. **p<0.01; ***p<0.001.

Original article: Oncotarget. 2017; 8:39087-39100. https://doi.org/10.18632/oncotarget.16598

