# Supplementary material for: Cytochrome P450 1B1 inhibition suppresses tumorigenicity of prostate cancer via caspase-1 activation
Source: Oncotarget. 2017 Mar 27;8(24):39087–100. doi: 10.18632/oncotarget.16598 (PMC5503597; doi:10.18632/oncotarget.16598)
Supplement: Supplementary file 1 [file oncotarget-08-39087-s001.pdf]

Cytochrome P450 1B1 inhibition suppresses tumorigenicity of prostate cancer via caspase-1 activation

SUPPLEMENTARY MATERIALS

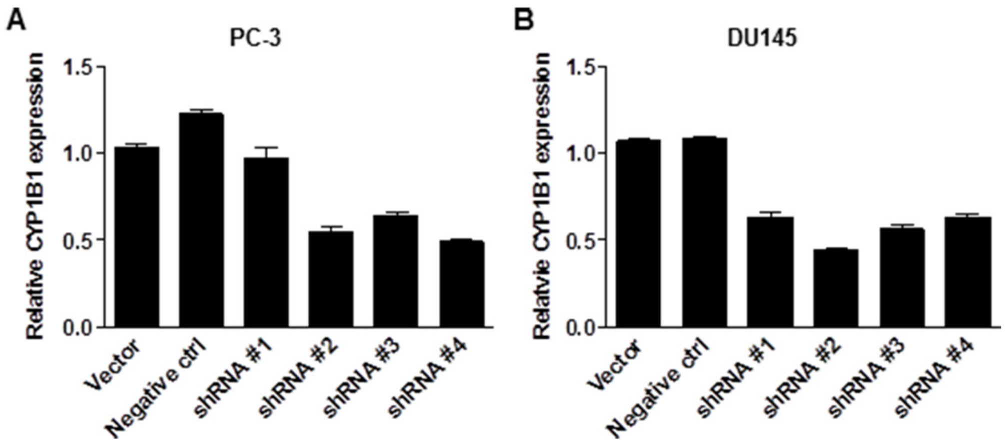

Supplementary Figure 1: Reduction of CYP1B1 mRNA expression by specific shRNA. After transient transfection of each shRNA construct, CYP1B1 mRNA expression in PC-3 **(A)** and DU145 **(B)** cells was determined by qRT-PCR.

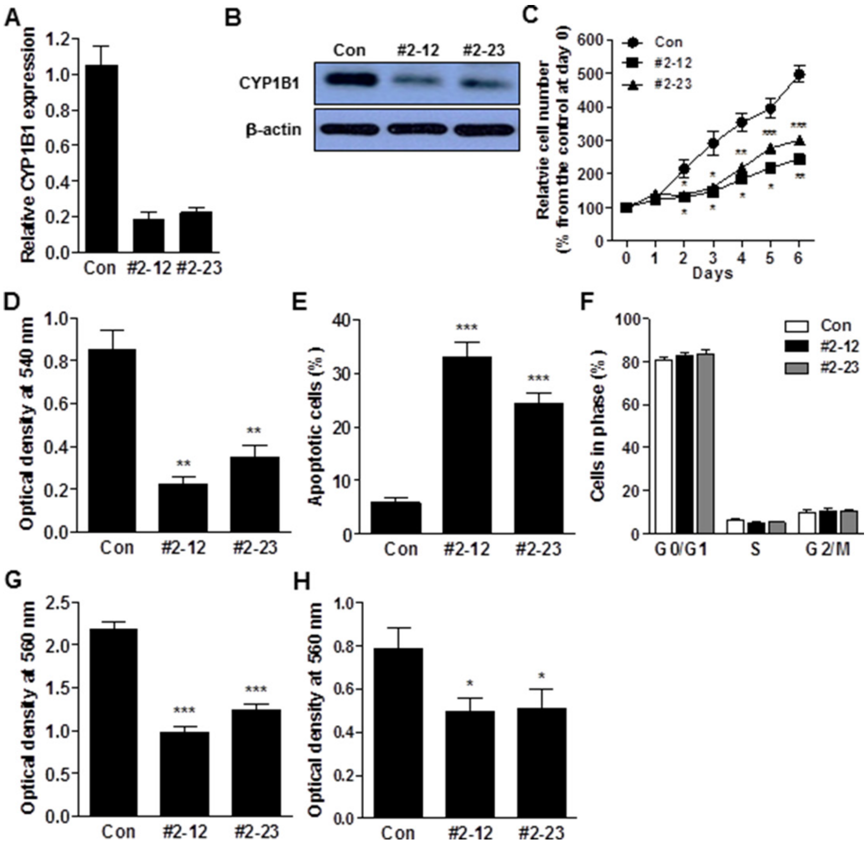

Supplementary Figure 2: CYP1B1 inhibition suppresses *in vitro* tumorigenicity in DU145 cells. (A and B) Expression of CYP1B1 mRNA (A) and protein (B) in CYP1B1 shRNA or control shRNA expressing DU145 cells. Levels were determined by qRT-PCR and Western blot, respectively. **(C to H)** Effect of CYP1B1 knockdown on *in vitro* tumorigenicity. Cell proliferation as determined by MTS assay at the indicated times (C). Colony formation as determined by crystal violet staining (D). Apoptotic cell death as examined by flow cytometric analysis using double staining with Annexin V-FITC and 7-AAD (E). Cell cycle progression as determined by DAPI staining (F). (G and H) Cell migration (G) and invasion (H) capability as determined by transwell migration and invasion assay, respectively. *p<0.05; **p<0.01; ***p<0.001.

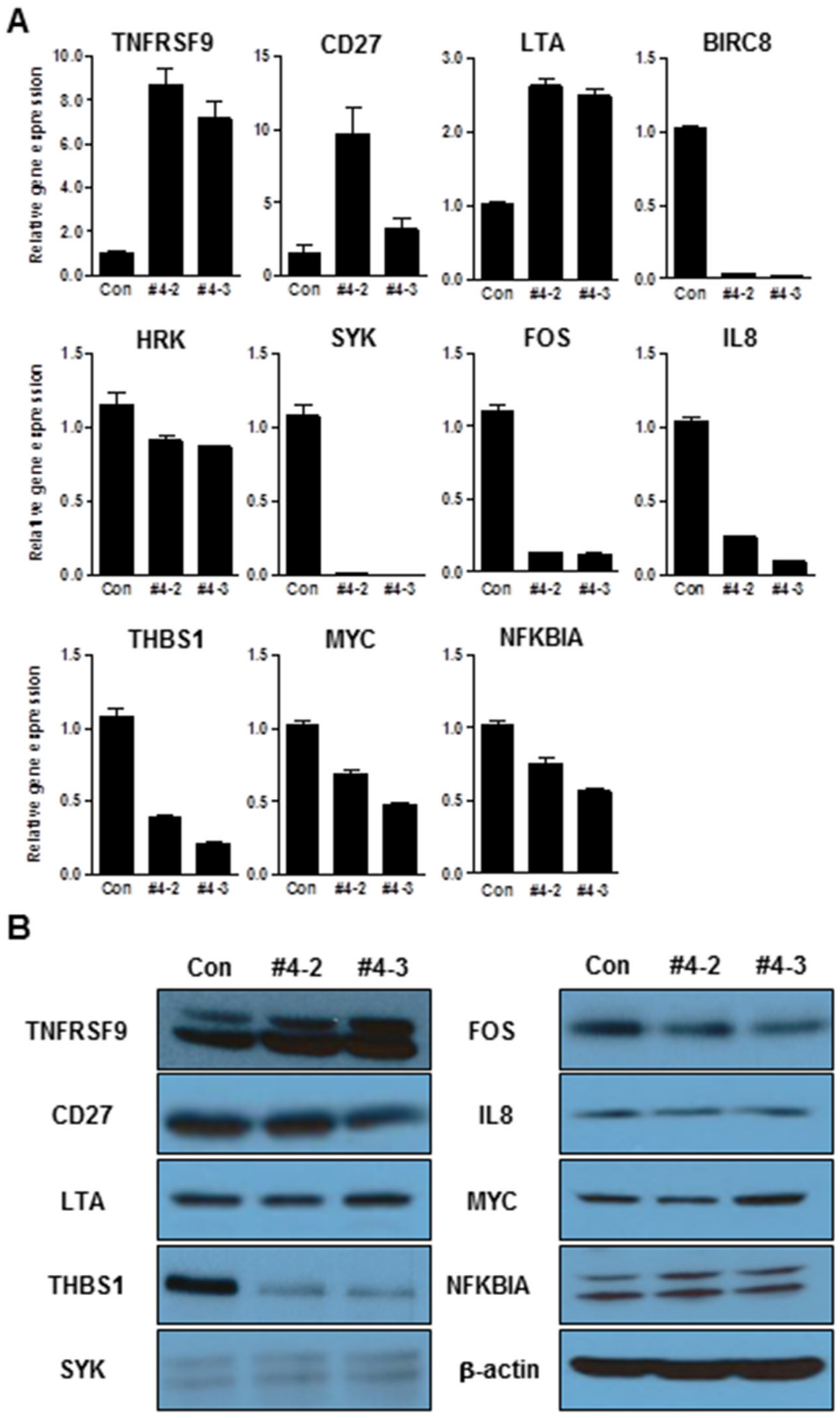

Supplementary Figure 3: Validation of candidate gene expression selected from PCR Arrays. (A and B) Candidate gene expression was examined by qRT-PCR (A) and Western blot (B).

Supplementary Table 1: Clinicopathologic characteristics of PCa patients

Pathological variables	Total (n=62), n (%)
**Age (years)**	
Mean	65
Range	45-84
**T-stage**	
pT2	31 (50.0)
pT3	26 (41.9)
pT4	5 (8.1)
**Gleason score**	
4-6	22 (35.5)
7	25 (40.3)
8-10	15 (24.2)
**Pathological diagnosis**	
Adenocarcinoma	62 (100.0)
